# Medical Society of North Carolina

**Published:** 1873-06

**Authors:** 


*Medical Society of North Carolina.*

This able body of medical gentlemen held its annual session last May. The session was interesting and harmonious. Drs. Satchwell and Norcom held a discussion on Acute Inflammation, which was the "big thing" of the session. A correspondent remarks: Dr. S. S. Satchwell, of New Hanover, began, and for two and a quarter hours held spell-bound with earnest attention not only the medical fraternity, but many others who had been attracted thither to hear the debate. As I listened, I could but think, if the Doctor's late address before the Alumni of New York College approximated this, how just and merited was the praise awarded, for rarely has it been my lot to enjoy such a literary treat, replete, as it was, with language most chaste, dialectics most sound, a "*chef d'euvre*" that the Doctor might well be proud of. Of the merit of Dr. Satchwell's address, certainly in a literary point of view the doctors even did not disagree, some going so far as to pronounce it the ablest production that had ever been delivered before their body.

Dr. Norcom's reply is said to have been an able exposition of his views on the subject.

Dr. Thomas F. Wood, the orator, delivered an address replete with noble sentiments and enlarged views, on the "Science of Medicine." Dr. Whitehead, the retiring President, delivered a chaste and able valedictory. Dr. Norcom was chosen President; Dr. James McKee, of Raleigh, Secretary; and Dr. Pearce, Orator.

Prominent among the actors were Drs. Satchwell and Wood, of New Hanover; Dr. C. T. Murphy, of Sampson; Dr. Hyatt, of Lenoir; Dr. O. Hagan, of Pitt; Dr. Hines, of Raleigh; Dr. Foote, of Warren; Dr. Whitehead, of Rowan, and Dr. J. B. Jones, of Charlotte.

Thus ended one of the largest and pleasantest meetings of this Society, to meet again in Charlotte in May next.

